# Effects of Word Width and Word Length on Optimal Character Size for Reading of Horizontally Scrolling Japanese Words

**DOI:** 10.3389/fpsyg.2016.00127

**Published:** 2016-02-16

**Authors:** Wataru Teramoto, Takuyuki Nakazaki, Kaoru Sekiyama, Shuji Mori

**Affiliations:** ^1^Division of Cognitive Psychology, Faculty of Letters, Kumamoto UniversityKumamoto, Japan; ^2^Mitsubishi Electric CorporationTokyo, Japan; ^3^Department of Informatics, Faculty of Information Science and Electrical Engineering, Kyushu UniversityFukuoka, Japan

**Keywords:** reading, Japanese, scrolling, character size, word perception

## Abstract

The present study investigated, whether word width and length affect the optimal character size for reading of horizontally scrolling Japanese words, using reading speed as a measure. In Experiment 1, three Japanese words, each consisting of four Hiragana characters, sequentially scrolled on a display screen from right to left. Participants, all Japanese native speakers, were instructed to read the words aloud as accurately as possible, irrespective of their order within the sequence. To quantitatively measure their reading performance, we used rapid serial visual presentation paradigm, where the scrolling rate was increased until the participants began to make mistakes. Thus, the highest scrolling rate at which the participants’ performance exceeded 88.9% correct rate was calculated for each character size (0.3°, 0.6°, 1.0°, and 3.0°) and scroll window size (5 or 10 character spaces). Results showed that the reading performance was highest in the range of 0.6° to 1.0°, irrespective of the scroll window size. Experiment 2 investigated whether the optimal character size observed in Experiment 1 was applicable for any word width and word length (i.e., the number of characters in a word). Results showed that reading speeds were slower for longer than shorter words and the word width of 3.6° was optimal among the word lengths tested (three, four, and six character words). Considering that character size varied depending on word width and word length in the present study, this means that the optimal character size can be changed by word width and word length in scrolling Japanese words.

## Introduction

Reading is one of the most important functions facilitating intellectual activities in our daily life. Text for reading is provided statically by printed materials such as books and newspapers, and also dynamically by electronic devices such as the Times Square moving news display. The latter is, in general, termed scrolling (or drifting) text presentation, where a line of text drifts from right to left (or from bottom to top) along a single line. It is useful, especially when a large amount of information is to be displayed in a limited space such as train and building walls, websites, smartphones, and digital signage displays. Several previous studies have investigated the properties of the visual mechanisms underlying reading performance with the scrolling text method, and have reported key parameters affecting reading text such as character size (Legge et al., 1985), contrast (Legge et al., 1987), spatial frequency (Legge et al., 1985), the number of characters simultaneously visible on the screen (Legge et al., 1985), color, and luminance (Legge and Rubin, 1986).

Character size is highly important among the factors determining the legibility of text. Legge et al. (1985) investigated the influence of character size on reading speeds with the scrolling text method and reported that the maximum reading speeds were achieved over a 10-fold range of character sizes from 0.3° to 2.0°. Reading speeds slowed down rapidly as character size decreased below 0.2°, and also slowed down gradually for characters larger than about 2.0°. In particular, they referred to the smallest character size below which reading speeds begin to decline rapidly, as critical print size (CPS). CPS is the limit of character size at which people can read text at optimal speed, but not an acuity limitation to identifying characters (Legge, 2007). Legge et al. (1985, 1987, 1989), Akutsu et al. (1991), Mansfield et al. (1996), and Chung et al. (1998) measured CPS in several studies and reported that CPS for normally sighted readers was almost constant (about 0.2°) across different methods, although the overall reading speeds changed depending on the presentation method. Legge et al. (1989) compared the effect of character size on reading performance between scrolling and static text (i.e., printed text on paper) presentations. The results showed that CPS was 0.25° for both methods, but at 0.25° the difference in reading speed between them was a maximum of 250 words/min. Chung et al. (1998) used the rapid serial visual presentation (RSVP) paradigm (Rubin and Turano, 1992) to avoid the potential influence of oculomotor control and demonstrated that the average CPS across six subjects was 0.17°. Note that, while an average reading speed was 1171 words/min for RSVP text (Rubin and Turano, 1992), it was 250–300 words/min for scrolling text (Legge et al., 1985). These findings suggest that character size plays a crucial role in reading performance.

The present study further investigated the effect of character size on reading of scrolling text, focusing on word length (the number of characters in a word) and word width (visual angle of a word). Most of the previous studies used sentences as test stimuli. Thus, words of various lengths (and word width) were included in the test stimuli. From the viewpoint of eye movements in reading, it is well known that word length and word width are crucial factors for controlling saccade eye movements during reading of static text: fixation duration is longer for longer than shorter words (Just and Carpenter, 1980; Rayner et al., 1996; Calvo and Meseguer, 2002; Kliegl et al., 2004) and the number of fixations is larger for longer than shorter words (Rayner and McConkie, 1976; Rayner et al., 1996; Kliegl et al., 2004). Morrison and Rayner (1981) measured the saccade distance during reading of static sentences, while manipulating viewing distances. The results showed that the saccade distance was constant if measured in number of character spaces (5.3–5.7 character spaces, while the corresponding saccade size was 2.0° to 3.8°). It should be noted that the fixation duration increased with increasing viewing distance (i.e., a decrease of visual angle). McDonald (2006) investigated the genuine effect of word length on eye movements in reading, by presenting all words at the same visual angle within a sentence. They revealed that the fixation duration and the number of fixations increased with an increase in the number of characters in the word. Hautala et al. (2011) confirmed the findings of McDonald (2006) with their stimuli of a proportional font (Arial) and further showed that word width influenced other aspects of saccade eye movements, such as the fixation location in words and the probability of skipping a word. An increase in fixation duration and the number of fixations would normally be indicative of a decrease in reading speed. Thus, these findings suggest a possibility that word length and word width impact on reading speed. In fact, Legge et al. (1997, 2001) investigated the effect of word length on reading performance, using the RSVP paradigm, in order to clarify the relationship between reading speed and visual span, i.e., the number of characters that can be recognized without eye movements (O’Regan, 1990). Carver (1976) also pointed out the effect of word length on reading speed in terms of text difficulty (i.e., easier text generally includes shorter words, so reading speed is higher). These studies revealed that word length had an impact on reading performance of static text, especially at low contrast and in peripheral vision (Legge et al., 1997, 2001). However, no study has thus far investigated how word length and word width influence reading performance of scrolling text and, specifically, whether the character size effect previously observed in scrolling text is applicable for any word length and word width.

In Experiment 1, we investigated the effect of character size on reading speed in scrolling text presentation, using Japanese 4-character words written in Japanese Hiragana, which are similar in usage to the letters of English, but different in phonologic complexity (i.e., a syllable consists of a single letter in Hiragana, but one to several letters in English). In Experiment 2, we investigated whether the effect of character size observed in Experiment 1 was applicable for any word width and word length. The results showed that word width and word length substantially influenced the optimal character size in reading.

## Experiment 1

### Method

#### Participants

There were 10 participants (aged 20–28; all males). All the participants had normal or corrected-to-normal vision and were unaware of the purpose of the experiment. All were native Japanese speakers. This study followed the tenets of the Declaration of Helsinki, and the protocol was approved by the ethics committee of Faculty of Information Science and Electrical Engineering, Kyushu University. Written informed consent was obtained from all participants (including those of Experiment 2) prior to participation.

#### Experimental Design

A two-by-four within-participant factorial design was used: scroll window size (5 and 10 characters) and character size (0.3°, 0.6°, 1.0°, and 3.0°). The scroll window size was defined as character spaces in which a character moves on the screen from right to left. This is different from “scroll distance,” which is defined as character spaces in which a word moves, corresponding to the sum of the window size and word length. Since both scroll window size and scroll distance were based on character space, their visual angles varied depending on character size. Previous studies found that reading speeds increased with an increase in window size up to 4 (Legge et al., 1985) or 4.7 (Fine and Peli, 1996a) and were no further increase or decrease for the larger character sizes. By introducing, two types of scroll window size beyond five character spaces in the present study, we intended to confirm if the optimal character size observed in one scroll window size condition could be generalized to that in the other scroll window size. Note that character size, character space and character width were defined based on the center-to-center spacing of the adjacent characters throughout this paper^1^.

#### Apparatus and Stimuli

Visual stimuli were generated using a visual stimulus generator (ViSaGe; Cambridge Research Systems, UK), attached to an IBM-compatible personal computer (Dell PRECISION 390) and displayed on a γ-corrected 19-in. computer monitor (EIZO, Flex Scan T760) with a refresh rate of 100 Hz and a resolution of 1,024 × 768. All visual stimuli were presented in the center of the monitor.

All the words used were selected within the range of high word familiarity, i.e., over 5.4 (maximum: 7) in the Japanese familiarity-controlled word lists (Amano and Kondo, 2003). In each trial, test words were chosen randomly from a pool of 2,223 words in order to avoid the effect of context on reading performance (Fine and Peli, 1996b). All the words presented contained four Hiragana characters and four morae (phonological sound units that determine syllable weights) because the average word length in Legge et al. (1985) was 4.1 characters so our results would be comparable to their results using 4-character words. Moreover, we found in the preliminary experiment that 4-character words were appropriate, because they were not easily identified upon sight of the first one or two characters, but shorter character words were very easy to identify. Also, reading performance for words containing more than six characters was degraded drastically. The words were rendered in MS Gothic Japanese, a fixed-width font, and were presented as black characters (0.39 cd/m^2^) on a white background of 68.62 cd/m^2^.

#### Procedure

**FIGURE 1:**
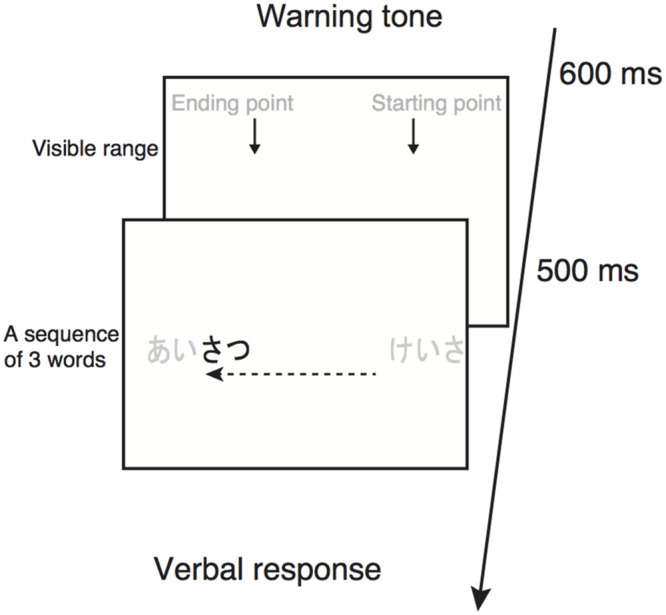
**Schematic illustration of experimental procedure in Experiments 1 and 2.** Gray-colored characters are not visible during the experiments. The arrows in the upper panel indicate the starting and ending points of scrolling text. The dotted arrow indicates the direction of scrolling.

Experiments were conducted with binocular viewing in a dark room. Immediately after the participant’s button press, a warning tone was presented for 600 ms, followed by a 500-ms presentation of two arrows indicating the starting and ending points of scrolling text (**Figure 1**). Soon after the disappearance of the arrows, three Japanese words were displayed consecutively^2^, one word at a time. Although the participants were able to look anywhere they wanted on the display before the presentation of the words because of no fixation point on the display, they normally looked around the starting point of scrolling. The participants were instructed to read the words aloud as accurately as possible, irrespective of the word order within the sequence. They were allowed to complete their verbalization during the presentation of words as well as after the words disappeared from the display. There was no time constraint for their responding. The experimenter assessed their responses and entered the number of correct responses into the computer. Immediately after that, a warning tone was presented for 600 ms and the next trial began.

The scrolling speed was manipulated by changing the exposure duration of a word on the monitor—the time taken from the appearance of the initial character until the disappearance of the fourth character. The initial exposure duration of each word was determined according to the performance of each participant in the preliminary trials so that it was sufficiently long (i.e., slow in scrolling speed) for each participant to report all the words. In subsequent trials, the exposure duration varied from trial to trial according to a staircase method (Cornsweet, 1962). The exposure duration was decreased when eight or nine words out of nine words (three words × the last three trials) were correctly reported (88.9% correct). The exposure duration was decreased by 80 ms for the first decrement, 40 ms for the second decrement, and 10 ms for subsequent decrements. Conversely, the exposure duration was increased by 10 ms when the number of words correctly reported was less than eight out of nine words. The staircase was terminated after eight reversals of the exposure duration sequence. The threshold of the exposure duration was calculated as the average of all but the first two reversals. We confirmed in the preliminary experiment that the last six reversals were enough to produce the stable threshold. Each participant performed one staircase per each character size per each scroll window size. The average number of trials they performed was 17.9 ± 3.4 (standard deviation). Reading speed in words per minutes (Legge et al., 1985; Carver, 1990) was also calculated, using the threshold (in ms), according to the following equation:

$$\mathrm{Reading}\;\mathrm{speed}\;(words/min)\;=\;\frac{60000}{\mathrm{threshold}\;\mathrm{exposure}\;\mathrm{duration}}$$

Thus, the reading speed was defined as the number of words readable for 1 min with 88.9% accuracy under a given condition. The optimal character size was defined as the character size with which reading performance reached the maximum speed.

To present each character clearly on the monitor, two viewing distances were adopted. The viewing distance was 172 cm for the character sizes of 0.3° and 0.6° and 57 cm for the character sizes of 1.0° and 3.0°. Different viewing distances were tested in different blocks. Half of the participants performed the 172-cm viewing distance block first and the other half performed the 57-cm viewing distance block first. The order of conditions tested within a block was randomized. It took about 80 min for each participant to complete all conditions.

### Results and Discussion

**FIGURE 2:**
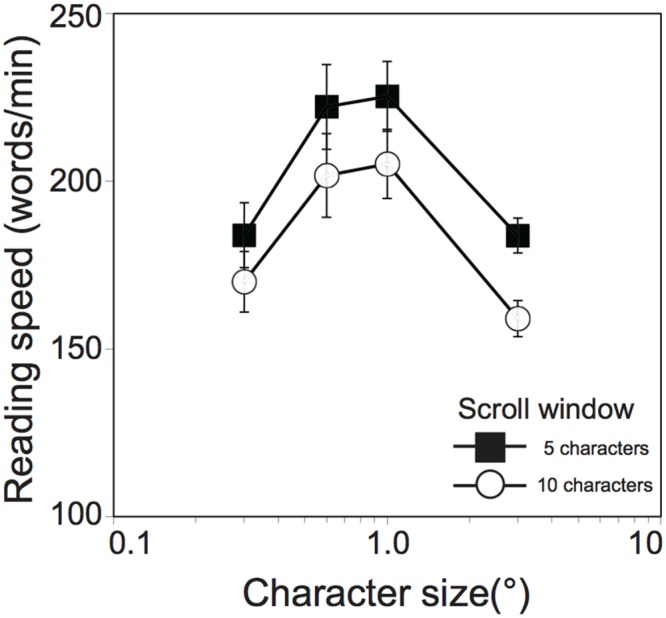
**Reading speed (ms/word) as a function of character size (°) in Experiment 1 (*N* = 10).** Filled squares and open circles indicate scrolling window size of 5 and 10, respectively. Error bars denote the standard error of the mean.

Threshold exposure durations obtained in Experiment 1 are shown in **Table 1**. Averages of calculated reading speeds across participants are shown as a function of character size in **Figure 2**. The reading speed increased with an increase in character size, reached a maximum with 1.0° of character size for both the 5-character (225 ± 10.4 words/min) and 10-character (205 ± 10.3 words/min) scroll window size conditions, and decreased for the larger character size. Since Shapiro–Wilk tests revealed that the data were normally distributed in all variables (*W* ≥ 0.881, *p* ≥ 0.132), the reading speeds were analyzed in a two-factor analysis of variance (ANOVA) with within-participants factors^3^ (scroll window size and character size). A main effect of scroll window size was significant [*F*(1,9) = 33.01, *p* < 0.001, $\eta_{G}^{2}$ = 0.103]: the reading was faster for the 5-character than for the 10-character scroll window size conditions. A main effect of character size was also significant [*F*(3,27) = 27.15, *p* < 0.001, $\eta_{G}^{2}$ = 0.458], but there was no significant interaction between them [*F*(3,27) = 1.97, *p* = 0.142, $\eta_{G}^{2}$ = 0.004]. A Tukey’s *post hoc* test for the character size factor (α < 0.05, *MSe* = 386.58) revealed that reading speed was significantly faster for the 0.6° and 1.0° than the 0.3° and 3.0° conditions. The optimal character size observed in this experiment are almost consistent with those of previous studies using scrolling English words (e.g., Legge et al., 1985; Akutsu et al., 1991), indicating that the effect of character size on reading performance can be generalized beyond differences in tested words (i.e., Japanese Hiragana words) and the experimental procedure. It should be noted that the maximum reading speeds (225 words/min and 205 words/min for the 5-character and 10-character scroll window size conditions, respectively) were slower than those of previous studies (about 300 words/min; Legge et al., 1985; Akutsu et al., 1991). This might be from numerous factors such as language (Japanese vs. English), context (random words vs. continuous sentences), experimental procedure (one word at a time vs. sentences), and so on.

**Table 1:** Average threshold exposure durations in Experiment 1(ms).

	Character size (°)			
Scroll window size (char.)	0.3	0.6	1.0	3
5	334 (52.6)	278 (48.4)	272 (39.6)	329 (29.3)
10	362 (60.5)	307 (55.4)	299 (45.3)	382 (43.2)

*Numbers in parenthesis represent standard deviations.*

**FIGURE 3:**
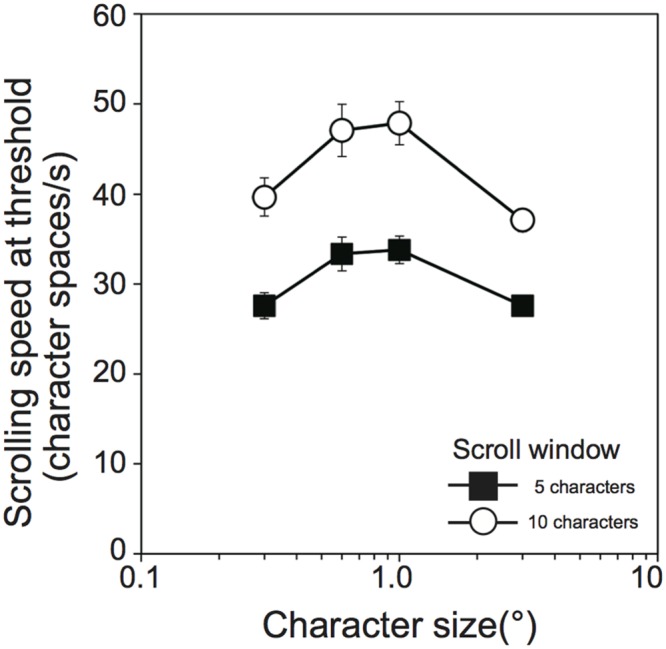
**Scrolling speed (character spaces/s) at threshold exposure duration as a function of character size (°) in Experiment 1 (*N* = 10).** Filled squares and open circles indicate scrolling window size of 5 and 10, respectively. Error bars denote the standard error of the mean.

Although the optimal character size was consistent between the scroll distance conditions, the overall reading speed was larger for the 5-character than for the 10-character scroll window size conditions. However, it should be noted that reading speeds in the present definition are derived only from the threshold exposure time. In the case where the threshold exposure time is identical between 5-character and 10-character scroll window size conditions, reading speed (words/min) would be the same while scrolling speed (°/s) would be higher for the 10-character than the 5-character conditions. This is because the exposure time measure disregards the difference in scroll distance. In order to appreciate the effect of scroll window size on reading performance, we computed a new reading performance measure, “scrolling speed at threshold,” according to the following equation:

$$\mathrm{Scrolling}\;\mathrm{speed}\;\mathrm{at}\;\mathrm{threshold}\;(\mathrm{character}\;spaces/s)\;=\frac{\left(\mathrm{scroll}\;\mathrm{window}\;\mathrm{size}\;+\;4\right)}{\left(\mathrm{threshold}\;\mathrm{exposure}\;duration/1000\right)}$$

where ‘4’ in the numerator of the right-hand side is the word length, i.e., four characters (see “Experimental Design”). Average scrolling speeds at thresholds across the participants are shown as a function of character size in **Figure 3**. The scrolling speed at threshold increased with an increase in character size, reached a maximum with 1.0° of character size for both the 5-character (33.8 ± 4.7 character spaces/s) and 10-character (47.9 ± 7.2 character spaces/s) scroll window size conditions, and decreased for the larger character size. Overall, the scrolling speeds were larger for the 10-character than 5-character scroll window size conditions. Since Shapiro–Wilk tests revealed that the data were normally distributed in all variables (*W* ≥ 0.881, *p* ≥ 0.132), a two-way ANOVA with within-participants factors (scroll window size and character size) was performed. The analysis revealed that both main effects and an interaction were significant [scroll window size, *F*(1,9) = 153.55, *p* < 0.001, $\eta_{G}^{2}$ = 0.539; character size, *F*(3,27) = 28.30, *p* < 0.001, $\eta_{G}^{2}$ = 0.306; scroll window size × character size, *F*(3,27) = 11.56, *p* < 0.001, $\eta_{G}^{2}$ = 0.024]. The analysis of the interaction revealed that maximum scrolling speeds were significantly larger for the 10-character than 5-character scroll window size conditions across all character size [0.3, *F*(1,36) = 114.54, *p* < 0.001, $\eta_{G}^{2}$ = 0.713; 0.6, *F*(1,36) = 148.26, *p* < 0.001, $\eta_{G}^{2}$ = 0.763; 1.0, *F*(1,36) = 155.42, *p* < 0.001, $\eta_{G}^{2}$ = 0.771; 3.0, *F*(1,36) = 71.30, *p* < 0.001, $\eta_{G}^{2}$ = 0.607]. This new analysis suggests that the slower reading speed in the 10- than 5-character scroll window size conditions could be also explained by the difference in scrolling speeds. Thus, to properly evaluate the effect of the scroll window size on reading performance, a more sophisticated word presentation method is necessary. Of importance in the present study is the fact that the reading performance was highest in the range of 0.6° to 1.0°, irrespective of the scroll window size tested.

## Experiment 2

Experiment 1 demonstrated that reading performance was highest in the range of 0.6° to 1.0°, irrespective of the scroll window size. The effect of scroll window size was also observed. Since Experiment 1 used only 4-character words, the word width was changed depending on the character size so that there could be an interaction between character size and word width. Thus, Experiment 2 investigated whether the optimal character size observed in Experiment 1 was applicable for any word width and word length. If character size is the only crucial factor for reading, reading performance should be highest in the range of 0.6° to 1.0° and degraded out of that range, irrespective of word width and word length.

### Method

#### Participants

There were 15 participants (aged 19–25; all males). All the participants had normal or corrected-to-normal vision and were unaware of the purpose of the experiment. All were native Japanese speakers.

#### Experimental Design, Stimuli, and Procedure

**FIGURE 4:**
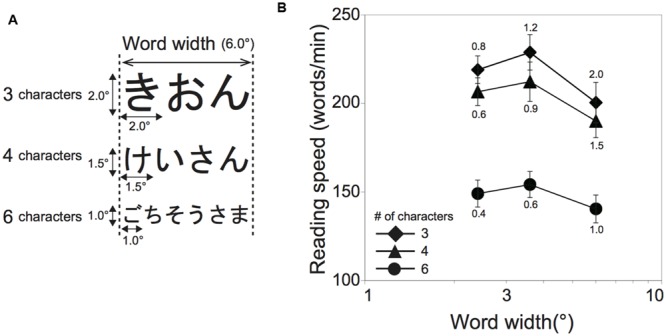
**Schematic illustrations of experimental stimuli and results of Experiment 2. (A)** The stimuli for the 6° word width condition are shown. **(B)** Reading speed (ms/word) is shown as a function of word width (°) in Experiment 2 (*N* = 15). Word length (number of characters) is designated by symbol type. The number added above or below each symbol is the character size for the condition. Error bars denote the standard error of the mean.

A three-by-three within-participant factorial design was used: word length (the number of characters in a word; three, four, and six characters) and word width (2.4°, 3.6°, and 6.0°). The character size was adjusted according to the word length and the word width (see **Figure 4A**). For example, the character sizes for three character words for 2.4°, 3.6°, and 6.0° of word width were 0.8°, 1.2°, and 2.0°, respectively, while those for four character words were 0.6°, 0.9°, and 1.5°, respectively, and those for six character words were 0.4°, 0.6°, and 1.0° respectively. If character size is the only crucial factor for reading, reading performance should be highest for 2.4° of word width in the 3-character word condition, for 2.4° and 3.6° of word width in the 4-character condition and for 3.6° and 6.0° of word width in the 6-character condition.

Three and four character words were selected within the range of high word familiarity, i.e., over 5.4 (maximum: 7) in the Japanese familiarity-controlled word lists, while six character words were selected within the range of word familiarity over 5.0 in the list because of fewer samples. In each trial, test words were chosen randomly from a pool of 1,518 words for three character words, 1,598 words for four character words, and 937 words for six character words. All the words were presented in Hiragana only. The words were rendered in MS Gothic Japanese, a fixed-width font, and were presented as black characters (0.86 cd/m^2^) on a white background of 105.13 cd/m^2^. The scroll window size was 10 character spaces across conditions.

To present each character clearly on the monitor, two viewing window sizes were adopted. The viewing window size was 172 cm for the character size under 0.8° and 57 cm for character size more than 0.8°. Different combinations of word length and word width were tested in different sessions. The order of conditions tested was randomized. Thus, the participants performed nine experimental sessions.

### Results and Discussion

Threshold exposure durations obtained in Experiment 2 are shown in **Table 2**. Averages of calculated reading speeds across participants are shown as a function of word width in **Figure 4B**. Each line indicates the number of characters in a word. The number added above or below each symbol was the character size for the condition. Shorter word lengths resulted in higher reading speeds for each word width. There was a peak of reading speed at the word width of 3.6°, irrespective of the number of characters in a word. Since Shapiro–Wilk tests revealed that the data were not normally distributed in a variable (*W* = 0.887, *p* = 0.011), we applied logarithmic transformation to the whole data (the normality test results after transformation: *W* ≥ 0.888, *p* ≥ 0.063). A two-factor ANOVA with within-participants factors (word length and word width) was performed on the transformed data. There were significant main effects of word length [*F*(2,28) = 84.91, *p* < 0.001, $\eta_{G}^{2}$ = 0.484] and word width [*F*(2,28) = 26.35, *p* < 0.001, $\eta_{G}^{2}$ = 0.074] and no significant interaction between them [*F*(4,56) = 0.64, *p* = 0.637, $\eta_{G}^{2}$ = 0.002]. Tukey’s *post hoc* tests for the word length factor (α < 0.05, *MSe* = 0.004) revealed that the reading speed was significantly different among these word length conditions: the 6-character condition obtained slower reading speed than the others. Notably, the *post hoc* tests for the word width factor (α < 0.05, *MSe* = 0.001) revealed that the reading speed for the 6.0° word width condition was slower than those for the other conditions. Experiment 1 showed the highest reading performance for the 0.6° and 1.0° character size conditions. However, the data of Experiment 2 demonstrated that reading performance became worse for the 1.0° than 0.4° character size condition if words extended to 6.0° wide and consisted of six characters. These results indicate that word width and word length have an impact on the optimal character size at least in the range of character size tested.

**Table 2:** Average threshold exposure durations in Experiment 2 (ms).

	Word width (°)		
Word length (char.)	2.4	3.6	6.0
3	279 (38.2)	269 (41.1)	311 (57.4)
4	296 (43.4)	293 (52.8)	326 (57.5)
6	416 (75.2)	402 (72.8)	445 (88.0)

*Numbers in parenthesis represent standard deviations.*

## General Discussion

The present study investigated the influence of word width and word length on the optimal character size for reading of horizontally scrolling Japanese words, by using reading speed as a measure. The results of Experiment 1 demonstrated that reading performance was highest at the character sizes of 0.6° to 1.0°, irrespective of the scroll window size (5-character and 10-character spaces). Experiment 2 investigated, how word width and word length influence the effect of character size observed in Experiment 1. The results showed that reading speed increased with a decrease in word length and that the reading performance became worse even for the character size of 1.0°, with which the highest reading performance was obtained in Experiment 1 if words extended to 6.0° wide and consisted of six characters. These findings suggest that both word width and word length can influence the optimal character size in reading scrolling text.

The effect of character size on reading has been intensively investigated. A common finding is that the maximum reading speed is obtained over a wide range of character sizes (0.2°–2.0° in Legge et al., 1985), and declines sharply below CPS and gradually for larger character sizes. The results of Experiment 1 are consistent with the previous studies: reading speed was nearly constant for 0.6° and 1.0° of character size, and declined for 0.3° and 3.0°. More important results of the present study are that the effect of character size can be dependent on word width and word length as shown in Experiment 2. Most of the previous studies used sentences as test stimuli so that words of various widths and lengths were included. Therefore, it was unclear whether these parameters can influence the effect of character size on reading. Experiment 2 showed that, for the condition in which words extended 6.0° wide and consisted of six characters, the reading performance became worse even for the 1.0° character size condition, which had yielded the maximum reading speed in Experiment 1. The effects of word length and word width on reading have already been reported in studies investigating eye-movements during reading. Longer words receive a longer fixation duration and a greater number of fixations than shorter words (Rayner and McConkie, 1976; Just and Carpenter, 1980; Rayner et al., 1996; Calvo and Meseguer, 2002; Kliegl et al., 2004). These findings imply that word width and word length can have significant impacts on reading speed as well, because the fixation duration and the number of fixations are closely related to reading speed. The present study provides evidence that word width and word length can influence not only eye movements in reading but also reading speed.

The effects of word width and word length observed in the present study demand an explanation that deals with the visual processing of character strings rather than individual characters (i.e., character acuity, contrast sensitivity, and so on). We speculate that concepts of visual span (Legge et al., 2001) and uncrowded span (Pelli et al., 2007) can explain our findings. Legge et al. (2001) developed a psychophysical method (trigram method) to measure visual span, the number of adjacent characters that can be read without moving eyes. Specifically, they presented strings of three characters at several positions left and right of the fixation, so briefly that no eye movements could occur. The participants were asked to report all three characters in left-to-right order. Character recognition accuracies (percent correct rates) were plotted as a function of distance of the fixation. The size of the visual span was defined as the distance from the fixation for which character recognition accuracy exceeded 80% correct. Legge et al. (2001, 2007) and Yu et al. (2007, 2010) found a high correlation between reading speed and the size of the visual span. This indicates that visual span could be linked to reading speed. However, it was not clear what determined the size of the visual span. Pelli et al. (2007) introduced a concept of “crowding” of character recognition (Bouma, 1970, 1973) into reading and demonstrated that crowding imposes the major limitation of the size of the visual span (see also Levi et al., 2007). In other words, they suggest that it is not character size but spacing between characters that limits the size of the visual span, and, as a result, reading speed. The critical character spacing, the minimum center-to-center spacing between target and flankers with which the target character can be identified at a threshold level, is proportional to eccentricity and extends 0.1° in the normal fovea (Bouma, 1970). Recognition of characters is prerequisite for recognition of words. Thus, when more than one character fall within a critical character spacing, not only character recognition but also word recognition is spoiled. The important point is that the critical character spacing is not uniform across a word. Words extend horizontally (and sometimes vertically) so they have several critical character spacings. Thus, there is a high possibility that more eccentric characters exceed the critical character spacing, especially within longer and wider words. This would be likely to explain our results in Experiment 2, where the reading performance for longer and wider words became worse even for the 1.0° character size condition, which had yielded maximum reading speed in Experiment 1.

## Conclusion

The present study investigated the influence of word width and word length on the optimal character size for reading of horizontally scrolling words. Results showed that the reading performance became worse even for the character size of 1.0°, with which the highest reading performance was obtained with four character words, when the presented words extended to 6.0° wide and consisted of six characters. These findings suggest that both word width and word length can influence the optimal character size in reading scrolling Japanese text at least.

## Author Contributions

All authors listed, have made substantial, direct and intellectual contribution to the work, and approved it for publication.

## Conflict of Interest Statement

The authors declare that the research was conducted in the absence of any commercial or financial relationships that could be construed as a potential conflict of interest.

## Footnotes

1 As a reference, we measured inter-character white space by using a maximum width character. The proportion of lateral white spacing of the character-to-character size is about 0.03°. That is, in the case where a character with a size of 1.0° is used, each of its left and right white spaces is 0.03° and the width of character *per se* is 0.94°.

2 We did not control the overlapping of phonemes and syllables in three consecutive words in each trial. The sequence of presented words was randomly selected from a large word list in each participant and the same word did not appear until the list was completed. Thus, we think that the overlap effect on the whole reading performance would be very small, if any.

3 In this and the subsequent ANOVAs, the significant levels were computed using Hyunh–Feldt corrected degrees of freedom for lack of sphericity in repeated measures.
